# What Keeps Older Adults Moving? An Analysis of Barriers and Motivation Across Different Exercise Settings

**DOI:** 10.1007/s10823-026-09580-1

**Published:** 2026-05-22

**Authors:** Bruno Fernando de Souza Tavares, Eduardo Quadros da Silva, Grazieli Covre da Silva, Sonia Maria Marques Gomes Bertolini, José Roberto Andrade do Nascimento, Daniel Vicentini de Oliveira

**Affiliations:** 1 Health Promotion Departament, Cesumar University, Maringá, Paraná Brazil; 2 Morphological Sciences Departament, Maringá State University, Maringá, Paraná Brazil; 3 Operational Human Performance Department, Air Force University, Rio de Janeiro, Rio de Janeiro Brazil; 4 Department of Human Movement Sciences, Maringá State University, Colombo Avenue 5790, Maringá, Paraná CEP 87020-900 Brazil

**Keywords:** Aging, Older adults, MotivationExercise

## Abstract

Perceived barriers and motivational factors may influence the adherence of older adults to physical exercise. Considering these variables, this study investigated the association between barriers and motivation for physical exercise among older adults. This cross-sectional study included 225 older adults engaged in physical exercise at private gyms, senior fitness centers, and sports centers in Maringá, Paraná, Brazil. The Exercise Motivation Inventory (EMI-2) and the Questionnaire on Barriers to Physical Activity Practice in Older Adults (QBPAFI) were used. Statistical analyses included descriptive statistics, Pearson’s correlation, multiple regression, and cluster analysis. Results indicated that older adults attending sports centers reported higher perceptions of physical, social, and belief-related barriers, as well as lower motivation for physical condition and social recognition, compared to those from gyms and Senior Fitness Academies (*p* < 0.05). Regression analyses revealed that higher weekly exercise duration was the strongest predictor of motivation, and higher frequency was associated with lower belief-related barriers. Women reported more external barriers, while men were more motivated by competition (*p* < 0.05). Cluster analysis revealed two distinct profiles: Cluster 1 (“low motivation and moderate perception of barriers,” *n* = 78) and Cluster 2 (“high motivation and low perception of barriers,” *n* = 147). Individuals in Cluster 2 reported more weekly exercise hours (*p* = 0.002), suggesting that higher motivation and lower perceived barriers are linked to greater adherence. Social and motivational barriers negatively affect adherence, while belief-related barriers may serve as incentives for disease prevention.

## Introduction

Population aging is a global phenomenon that significantly reshapes social, economic, and healthcare dynamics. According to United Nations projections, the number of individuals aged 65 and over is expected to exceed 1.5 billion by 2050, underscoring the urgent need for effective strategies to promote health and quality of life in this population (Rosenberg et al., 2022). Regular physical exercise is a well-established protective factor in this context, contributing to disease prevention, functional autonomy, and mental well-being (Izquierdo et al., 2021).

Despite its recognized benefits, adherence to physical activity among older adults remains a persistent challenge, influenced by personal, social, and environmental factors (Cahalin et al., 2023; Suryadi et al., 2024). Common barriers include physical limitations, fear of injury, and insufficient knowledge about the benefits of exercise (Sun et al., 2024), which are further exacerbated in contexts with limited infrastructure, safety, and accessibility (Nau et al., 2019).

A promising framework for understanding these dynamics is Self-Determination Theory (SDT), which posits that human motivation is shaped by the degree to which social contexts satisfy three basic psychological needs: autonomy (feeling of volition and choice), competence (feeling effective and capable), and relatedness (feeling connected and supported by others) (Ryan & Deci, 2020). Exercise settings that fail to support these needs—limiting individual agency, providing insufficient instruction, or offering few opportunities for social connection—can undermine motivation and adherence. Conversely, environments that foster autonomy, build competence through progressive feedback, and encourage social bonds are more likely to sustain engagement in physical activity (Teixeira et al., 2012).

The characteristics of the exercise environment are therefore critical in shaping motivation. Public spaces—such as parks and community centers—often enhance accessibility and foster social interaction but may suffer from inadequate maintenance and perceived safety risks (Zhou et al., 2021). Private settings—such as fitness centers and specialized gyms—offer structured programs and professional guidance, but they may be financially or socially inaccessible for some populations (Dolenc et al., 2023). Moreover, the presence of qualified professionals has been shown not only to improve safety and outcomes but also to enhance motivation by promoting competence and relatedness (Buriticá-Marín et al. 2023; Zwingmann et al. 2024a). Supervised exercise programs, particularly those conducted outdoors, have demonstrated specific benefits for both physical and cognitive health (Zwingmann et al. 2024b).

The distinction between outdoor and indoor settings adds another layer of complexity. Outdoor activities, often associated with exposure to nature, are linked to improved mood and increased enjoyment (Wicks et al., 2022). Indoors offer predictability, safety, and access to equipment, enhancing perceived competence (Nau et al., 2019). Both environments, however, present limitations: outdoor spaces may be hindered by adverse weather or uneven terrain (Kilgour et al., 2024), while indoor settings may lack spontaneous social interaction and contact with natural elements (Frahsa, Hahn, & Thiel, 2024).

Although previous studies have investigated the influence of environment, supervision, or physical setting on older adults’ exercise behavior, these factors have mostly been explored in isolation (Huang et al., 2024; Barnett et al., 2017). Furthermore, existing research rarely integrates these contextual variables with SDT, limiting our understanding of how environmental conditions facilitate or hinder the satisfaction of basic psychological needs and behavior.

This study seeks to fill that gap by examining how different exercise practice profiles—defined by combinations of setting (public or private), supervision (with or without a professional), and environment—relate to older adults’ perceived barriers and motivation to exercise. We also investigate whether these motivational and barrier profiles are associated with the frequency and duration of weekly exercise, variables known to influence long-term adherence, but still understudied in terms of environmental and psychological factors.

By addressing these gaps, the study contributes to the literature by analyzing behavioral, motivational, and contextual factors in later-life exercise participation. It aims to inform the design of more effective and tailored interventions and public health policies that support sustainable physical activity engagement among aging populations.

## Methods

### Study Design and Ethical Aspects

This research is a quantitative, analytical, observational, and cross-sectional study, developed by the Strengthening the Reporting of Observational Studies in Epidemiology (STROBE) guidelines. The project was submitted to the Research Ethics Committee of the Metropolitan University Center of Maringá (Unifamma) and approved under opinion number 2.305.331.

### Participants

In 2022, the population of Maringá (Brazil) was 409,657 inhabitants, with projections estimating 425.983 people in 2024, of whom approximately 75.004 were aged 60 years or older. A non-probabilistic sample was intentionally and conveniently selected, consisting of 225 older adults who regularly practiced physical activity: 75 from private gyms, 75 from Senior Fitness Academies, and 75 from municipal sports centers, all located in Maringá, Paraná State, Brazil.

The inclusion criteria were: older adults of both sexes, aged 60 years or older, who self-reported practicing one or more types of physical activity (e.g., gymnastics, dance, water aerobics, stretching, strength training, Pilates Method exercises, among others) for at least six months in the exact location. Private gyms, municipal sports centers, or Senior Fitness Academies could offer these activities. Additionally, participants needed adequate verbal and auditory communication skills to complete the research questionnaires.

In 2024, Maringá had 76 Senior Fitness Academies, 14 municipal sports centers, and 171 private gyms (Prefeitura de Maringá, 2024). The data collection sites were selected through simple random sampling based on official registries. The list of private gyms was obtained from the Associação Comercial e Empresarial de Maringá (ACIM); the list of municipal sports centers was provided by the Municipal Department of Sports and Leisure; and the list of Senior Fitness Academies was obtained from the Municipal Health Department. Four locations were randomly selected from each list to ensure geographic distribution across different city regions.

There was no predefined number of locations initially contacted, as the sample was intentionally designed based on logistical feasibility and the goal of ensuring geographic diversity within the city. The choice of four locations per exercise setting (private gyms, municipal sports centers, and Senior Fitness Academies) was determined considering operational aspects such as researcher availability, time constraints, and accessibility, as well as the need to reach the intended number of participants (75 per group).

To screen for cognitive impairment and ensure participants’ ability to respond to the questionnaires, the Mini-Mental State Examination (MMSE) was administered. The first part evaluates orientation, memory, and attention (maximum score: 21), while the second part assesses specific skills, including naming and comprehension (maximum score: 9), for a total score of 30. The cutoff scores vary based on educational level:

- 20 points for illiterate individuals,
- 25 for 1–4 years of schooling,
- 26.5 for 5–8 years,
- 28 for 9–11 years, and.
- 29 for more than 11 years of education (Folstein et al., 1975; Brucki et al., 2003).

Exclusion criteria included older adults who practiced exercise exclusively with personal trainers, those with cognitive impairment identified by the MMSE, and those who engaged in physical activity in multiple settings (e.g., private gyms and Senior Fitness Academies). The exclusion of individual training with personal trainers aimed to maintain consistency in the type of exercise guidance across groups since personal training represents a highly individualized and tailored form of supervision that differs substantially from the group-based or unsupervised settings examined in this study. Similarly, participants who exercised in more than one type of environment were excluded to ensure that the motivational and barrier profiles could be attributed to a specific practice context, thus preserving the internal validity of group comparisons.

### Instruments

The authors developed a questionnaire to identify the sociodemographic profile and patterns of physical exercise practice. It included questions related to age, age group, sex, education level, monthly income (in minimum wage units), marital status, overall physical exercise practice duration, and the frequency and duration of specific types of physical activity practiced weekly.

The Exercise Motivations Inventory – EMI-2 was used to assess motivation for adherence to regular physical exercise (Guedes, Legnani, & Legnani, 2012). This instrument aims to identify, measure, and rank both intrinsic and extrinsic motivational factors related to physical activity. It consists of 44 items distributed across ten factors: fun/well-being (“Because it is rewarding in itself”), stress control (“To help manage stress”), social recognition (“To demonstrate my value to other people”), affiliation (“To make new friends”), competition (“Because I feel good competing”), health/rehabilitation (“To feel healthy”), disease prevention (“To avoid health problems”), body weight control (“To maintain body weight”), physical appearance (“To look younger”), and physical condition (“To develop muscles”). The items are answered using a 6-point Likert scale (0 = “not at all true” to 5 = “completely true”), headed by the statement “Personally, I practice (or could come to practice) physical exercise” (Guedes, Legnani, & Legnani, 2012). The values of each factor are obtained from the arithmetic mean of the values answered in their respective items. The validation study showed Cronbach’s alpha coefficients to be acceptable (0.738 to 0.918), and 78.4% of items had a substantial kappa index of agreement (> 61%) when the questionnaire was applied repeatedly.

The Questionnaire on Barriers to Physical Activity in Older Adults (QBPAFI) was used to assess perceived barriers to greater adherence to physical exercise. This questionnaire presents a list of 22 potential barriers, and participants are asked to indicate how often each barrier occurs using a 5-point Likert scale: 1 (Always), 2 (Often), 3 (Sometimes), 4 (Rarely), 5 (Never). Items can be distributed across five barriers: Physical (“I have no energy”), Social (“I am very shy or embarrassed”), Beliefs (“I do not believe that physical activity is good”), Motivation (“I need to rest and relax in my free time”), and External (“Lack of company”). The values of each factor are obtained from the arithmetic mean of the values answered in their respective items. The scaling method allows for the quantitative assessment of perceived barriers, providing greater precision in identifying the significance of each barrier (Hirayama, 2006).

### Procedures

Initially, the Municipal Department of Sports of Maringá was contacted to request authorization to conduct research at local sports centers. Likewise, authorization was sought from the Municipal Health Department to collect data at the Senior Fitness Academies. Four sports centers and four Senior Fitness Academies distributed across the city were randomly selected. For data collection in private gyms, authorization was first requested from the respective facility administrators. Four private gyms in the municipality were randomly selected to participate in the study.

Once the necessary permissions were obtained, those responsible for the sports centers and private gyms were asked to provide information regarding the days and times when group activities for older adults were offered. In private gyms specifically, schedules for times when older adults most frequently exercised individually were also requested. For Senior Fitness Academies, public spaces without a fixed activity schedule were randomly selected, based on the researcher’s availability, on different days and times.

The researcher approached older adults during visits and explained the study’s objectives and procedures to them. Those who agreed to participate signed the Informed Consent Form (ICF). Data collection took place between July and December 2024.

The questionnaires were administered collectively to participants who did not require assistance, with each session lasting approximately 30 to 45 min. For illiterate participants, individual appointments were scheduled to read and explain the objectives, questionnaires, and the ICF. These individual sessions lasted approximately 80 min to complete.

### Data Analysis

The analysis was conducted using both descriptive and inferential statistical approaches. In the descriptive analysis, absolute and relative frequencies were calculated for categorical variables, while means and standard deviations were used to measure central tendency and dispersion for numerical variables. For numerical variables, normality was assessed using the Kolmogorov-Smirnov test and by evaluating skewness and kurtosis coefficients.

Additionally, bootstrapping procedures (1,000 resamples; 95% BCa confidence interval) were applied to improve the reliability of the results, correct for potential deviations from normal distribution, account for unequal group sizes, and generate 95% confidence intervals for the means (Haukoos & Lewis, 2005).

One-way ANOVA was used to compare barrier perception and motivational factors based on variables related to physical activity practice, followed by Tukey’s post hoc test (for more than two groups). Additionally, 15 multiple linear regression models were conducted using the enter method to investigate the association between age, gender, weekly frequency of exercise, and weekly duration of exercise (independent variables) and the perceived barriers (Models 1 to 5) and motivational factors (Models 6 to 15) (dependent variables). Variance inflation factors (VIFs) were calculated to check for multicollinearity (VIF < 5.0).

Older adults were also grouped and classified using hierarchical and non-hierarchical cluster analyses based on barrier perception and motivation scores. First, a nearest-neighbor hierarchical cluster analysis was conducted, using squared Euclidean distance as the dissimilarity measure. The R² value was used to determine the number of clusters to retain. Based on this analysis, three clusters were identified. A non-hierarchical cluster analysis was performed to validate and classify individuals into these three retained clusters.

According to Cumming and Duda’s (2012) criteria, z-scores below − 0.5 were considered low; z-scores between − 0.5 and + 0.5, moderate; and z-scores above + 0.5, high. Chi-square tests were used to investigate associations between barrier perception and motivational profiles about physical activity variables. A significance level of *p* < 0.05 was adopted. Data analysis was conducted using SPSS software, version 29.0 (IBM Corporation, 2025).

## Results

A total of 225 older adults participated in the study, including 194 women and 31 men aged between 60 and 88 (M = 69.26; SD = 6.42). The participants exercised in private gyms (*n* = 75), Senior Fitness Academies (*n* = 75), and municipal sports centers (*n* = 75).

The majority of participants were aged 60 to 69 years (54.2%), lived with a partner (56.4%), had a monthly income between one and two minimum wages (50.7%), and rated their health as good (72.4%). Regarding education level, 35.6% reported being illiterate or having incomplete elementary education, 36.4% had completed elementary education, and 28.0% had completed higher education.

Table 1 presents the descriptive analysis of physical exercise variables, perceived barriers, and motivational factors related to older adults’ participation in physical exercise. Firstly, 50.2% of the participants reported exercising weekly, with 75.8% engaging in physical activity for 1 to 5 h per week. In general. Further, the participants reported the highest perception of barriers about external barriers (M = 14.11; SD = 4.25), followed by physical barriers (M = 8.76; SD = 3.21), motivational barriers (M = 6.67; SD = 2.42), social barriers (M = 6.44; SD = 2.58), and belief-related barriers (M = 5.72; SD = 1.28). Regarding motivational factors, the highest mean score was found for weight control (M = 4.15; SD = 0.84), followed by stress management (M = 4.01; SD = 0.90), disease prevention (M = 3.91; SD = 0.83), health rehabilitation (M = 3.82; SD = 1.15), fun/enjoyment (M = 3.85; SD = 0.89), competition (M = 3.80; SD = 1.07), affiliation (M = 3.70; SD = 1.07), appearance (M = 3.68; SD = 0.99), physical condition (M = 3.64; SD = 1.00), and social recognition (M = 3.61; SD = 1.04).

**Table 1:** Descriptive analysis of physical exercise variables, barriers, and motivational factors for physical exercise practice among older adults

Variables	Mean/*n*	SD/%	Skewness	Kurtosis
Weekly frequency of physical exercise practice				
Daily	99	44,00	**-**	**-**
Weekly	113	50,20	**-**	**-**
Rarely	13	5,80	**-**	**-**
Weekly duration of physical exercise				
Less than 1 h	36	16,10	**-**	**-**
1 to 3 h	99	44,40	**-**	**-**
3 to 5 h	70	31,40	**-**	**-**
More than 5 h	18	8,10	**-**	**-**
Barriers to Physical Activity Practice				
Physical	8.76	3.21	0.50	-0.15
Social	6.44	2.58	0.45	-0.84
Beliefs	5.72	1.28	-0.31	1.74
Motivation	6.67	2.42	0.47	-0.35
External	14.11	4.25	0.27	-0.21
Motivational Factors				
Disease prevention	3.91	0.83	-0.59	-0.25
Physical condition	3.64	1.00	-0.51	-0.54
Weight control	4.15	0.84	-0.96	-0.50
Appearance	3.68	0.99	-0.49	-0.53
Stress control	4.01	0.90	-0.92	0.34
Fun	3.85	0.89	-0.67	-0.21
Affiliation	3.70	1.07	-0.50	-0.78
Health rehabilitation	3.82	1.15	-0.84	-0.21
Competition	3.80	1.07	-0.72	-0.33
Social recognition	3.61	1.04	-0.51	-0.70

SD: standard deviation

Table 2 compares barriers and motivational factors for physical exercise among older adults based on their exercise practice setting. Significant differences were found between groups in physical barriers (*p* = 0.001), social barriers (*p* = 0.002), and belief-related barriers (*p* = 0.039), as well as in the motivational factors of physical condition (*p* = 0.026), competition (*p* = 0.041), and social recognition (*p* = 0.031). Notably, older adults who exercised at municipal sports centers reported a higher perception of physical and social barriers than those attending private gyms and Senior Fitness Academies. Additionally, sports center users reported a greater perception of belief-related barriers and lower motivation regarding physical condition and social recognition when compared to gym users. Finally, gym users showed greater motivation for competition than participants from Senior Fitness Academies.

**Table 2:** Comparison of barriers and motivational factors for physical exercise among older adults based on the place of exercise practice

VARIABLES	Place of physical exercise practice			*p*
	Gym (*n* = 75)	SFA (*n* = 75)	Sports Center (*n* = 75)	
	M (SD)	M (SD)	M (SD)	
Barriers to Physical Activity Practice				
Physical	8.24 (3.20)	8.19 (2.70)	9.85 (3.44)^a^	0.001*
Social	5.79 (2.06)	6.29 (2.36)	7.24 (3.04)^a^	0.002*
Beliefs	5.49 (1.22)	5.65 (1.22)	6.01 (1.33)^b^	0.039*
Motivation	6.23 (2.11)	6.69 (2.27)	7.09 (2.77)	0.089
External	13.28 (3.92)	14.44 (3.77)	14.63 (4.64)	0.098
Motivational Factors				
Disease prevention	4.04 (0.80)	3.88 (0.86)	3.79 (0.82)	0.157
Physical condition	3.89 (0.97)^b^	3.56 (1.02)	3.47 (0.99)	0.026*
Weight control	4.28 (0.81)	4.06 (0.89)	4.09 (0.82)	0.219
Appearance	3.80 (0.94)	3.56 (1.06)	3.69 (0.97)	0.344
Stress control	4.16 (0.85)	3.97 (0.90)	3.89 (0.95)	0.179
Fun	3.95 (0.89)	3.81 (0.86)	3.78 (0.91)	0.464
Affiliation	3.76 (1.03)	3.62 (1.08)	3.71 (1.11)	0.725
Health rehabilitation	4.01 (0.99)	3.66 (1.29)	3.80 (1.14)	0.176
Competition	4.03 (0.81)^c^	3.59 (1.17)	3.76 (1.16)	0.041*
Social recognition	3.84 (1.02)^b^	3.58 (1.05)	3.40 (1.00)	0.031*

*Significant difference: p < 0.05 – One-Way ANOVA between: a) Sports Center and Gym & Senior Fitness Academies; b) Gym and Sports Center; c) Gym and Senior Fitness Academies.SFA: Senior Fitness Academies. M: mean. SD: standard deviation

Table 3 compares barriers and motivational factors among older adults based on their frequency of physical exercise per week. Significant differences were found between groups in belief-related barriers (*p* < 0.001), motivational barriers (*p* < 0.001), and external barriers (*p* = 0.023), indicating that older adults who reported rarely exercising perceived higher levels of external and motivational barriers and lower perception of belief-related barriers compared to those who exercised daily or weekly. Regarding motivational factors, significant differences were observed in disease prevention (*p* = 0.006), weight control (*p* = 0.003), appearance (*p* = 0.020), stress management (*p* = 0.010), affiliation (*p* = 0.038), and social recognition (*p* = 0.040). These findings suggest that older adults who reported rarely exercising exhibited lower motivation for weight control and stress management compared to those who reported exercising daily or weekly. Additionally, those who rarely exercised had lower motivation for disease prevention, appearance, affiliation, and social recognition when compared to those who exercised daily.

**Table 3:** Comparison of barriers and motivational factors for physical exercise practice among older adults based on weekly exercise frequency

VARIABLES	Weekly frequency of physical exercise practice			p
	Daily(*n* = 99)	Weekly(*n* = 113)	Rarely(*n* = 13)	
	M (SD)	M (SD)	M (SD)	
Barriers to Physical Activity Practice				
Physical	8.72 (3.34)	8.70 (3.20)	9.61 (2.14)	0.614
Social	6.55 (2.81)	6.27 (2.41)	7.15 (2.19)	0.435
Beliefs	5.88 (1.14)	5.72 (1.30)	4.46 (1.51)^a^	< 0.001*
Motivation	6.72 (2.56)	6.34 (2.11)	9.23 (2.42)^a^	< 0.001*
External	14.27 (4.84)	13.65 (3.50)	16.92 (2.29)^a^	0.023*
Motivational Factors				
Disease prevention	4.06 (0.80)	3.84 (0.79)	3.33 (1.04)^b^	0.006*
Physical condition	3.81 (1.05)	3.51 (0.95)	3.40 (1.00)	0.058
Weight control	4.23 (0.80)	4.16 (0.82)	3.40 (1.01)^a^	0.003*
Appearance	3.85 (1.00)	3.59 (0.94)	3.13 (1.08)^b^	0.020*
Stress control	4.15 (0.92)	3.96 (0.84)	3.36 (1.07)^a^	0.010*
Fun	3.94 (0.91)	3.81 (0.84)	3.41 (1.03)	0.105
Affiliation	3.87 (1.07)	3.61 (1.06)	3.15 (0.90)^b^	0.038*
Health rehabilitation	3.93 (1.17)	3.78 (1.13)	3.36 (1.01)	0.205
Competition	3.97 (1.04)	3.69 (1.08)	3.35 (1.06)	0.051
Social recognition	3.76 (1.07)	3.53 (0.99)	3.06 (0.97)^b^	0.040*

* Significant difference: p < 0.05 – One-Way ANOVA between: a) Rarely vs. Daily and Weekly; and b) Daily vs. RarelyM: mean. SD: standard deviation

When comparing barriers and motivational factors for physical exercise among older adults based on weekly exercise duration (Table 4), significant differences were found between groups for all motivational factors (*p* < 0.05). Specifically, older adults who reported engaging in 3 to 5 h of physical activity per week demonstrated higher motivation in the following factors: physical condition, stress management, weight control, appearance, enjoyment, affiliation, health rehabilitation, competition, and social recognition, compared to those who reported exercising less than 1 h or between 1 and 3 h per week. Additionally, those who exercised 3 to 5 h per week also showed greater motivation for disease prevention than those who exercised less than 1 h per week.

**Table 4:** Comparison of barriers and motivational factors for physical exercise among older adults based on weekly exercise duration

VARIABLES	Weekly duration of physical exercise				*p*
	Less than 1 h(*n* = 36)	1 to 3 h(*n* = 99)	3 to 5 h(*n* = 70)	More than 5 h(*n* = 18)	
	M (SD)	M (SD)		M (SD)	
Barriers to Physical Activity Practice					
Physical	8.75 (2.92)	8.90 (3.14)	8.87 (3.57)	7.50 (2.59)	0.388
Social	6.89 (2.59)	6.50 (2.51)	6.43 (2.77)	5.17 (1.98)	0.138
Beliefs	5.44 (1.50)	5.95 (1.22)	5.56 (1.25)	5.56 (1.20)	0.101
Motivation	7.36 (2.73)	6.56 (2.26)	6.77 (2.56)	5.72 (1.71)	0.111
External	15.64 (3.73)	13.73 (3.48)	14.16 (5.06)	12.94 (4.14)	0.068
Motivational Factors					
Disease prevention	3.62 (0.96)	3.72 (0.82)	4.31 (0.65)^a^	3.88 (0.72)	< 0.001*
Physical condition	3.38 (1.05)	3.32 (1.00)	4.14 (0.85)^b^	3.96 (0.61)	< 0.001*
Weight control	3.85 (0.95)	4.03 (0.88)	4.41 (0.68)^b^	4.31 (0.73)	0.003*
Appearance	3.24 (1.09)	3.47 (0.96)	4.12 (0.81)^b^	3.92 (0.89)	< 0.001*
Stress control	3.69 (1.05)	3.82 (0.96)	4.35 (0.65)^b^	4.26 (0.68)	< 0.001*
Fun	3.55 (1.06)	3.66 (0.89)	4.22 (0.71)^b^	3.98 (0.64)	< 0.001*
Affiliation	3.42 (1.14)	3.50 (1.13)	4.08 (0.92)^b^	3.85 (0.79)	0.002*
Health rehabilitation	3.59 (1.18)	3.57 (1.28)	4.20 (0.85)^b^	4.17 (0.89)	0.001*
Competition	3.46 (1.12)	3.61 (1.15)	4.18 (0.87)^b^	3.97 (0.85)	0.001*
Social recognition	3.18 (1.06)	3.36 (1.02)	4.13 (0.92)^b^	3.72 (0.65)	< 0.001*

* Significant difference: p < 0.05 – One-Way ANOVA between: a) 3 to 5 hours and less than 1 hour; b) 3 to 5 hours and less than 1 hour and 1 to 3 hours. M: mean. SD: standard deviation

To examine the predictors of perceived barriers and motivational factors for physical exercise, 15 multiple linear regression models were conducted. The independent variables tested in each model were age, gender, weekly frequency of exercise, and weekly duration of exercise. The results are detailed in Table 5.

The analysis of barriers revealed that the predictive models for Physical (M1), Social (M2), and Motivation (M4) barriers were not statistically significant, indicating that the set of predictors did not significantly explain the variance in these outcomes. However, the regression model for the Beliefs (M3) barrier was statistically significant (F = 3.835, *p* < 0.01, adjusted R^2^ = 0.050). Within this model, weekly frequency of exercise emerged as the sole significant predictor (B = − 0.540, 95% CI [− 0.845, − 0.235], *p*<0.01), indicating that a higher frequency of exercise per week is associated with a lower perception of barriers related to limiting beliefs. Similarly, the model for External Barriers (M5) was significant (F = 3.602, *p* < 0.01, adjusted R² = 0.045). In this model, only gender was a strong positive predictor (B = 1.980, 95% CI [0.410, 3.550], *p*<0.01), indicating that women perceive significantly more external barriers compared to men.

**Table 5:** Age, gender, and physical activity variables as predictors of the barriers and motivational factors among older adults

Dependent variables	Predictors				Adjusted *R*^2^	F	Durbin-Watson
	Age	Gender	Weekly frequency of exercise	Weekly duration of exercise			
	B (CI)	B (CI)	B (CI)	B (CI)			
Barriers							
Physical (M1)	-0,010 (-0,078; 0,057)	0.349 (-0.897; 1.595)	0.036 (-0.750; 0.823)	-0.218 (-0.781; 0.345)	0.010	0.312	1.542
Social (M2)	-0.005 (-0.058; 0.049)	0.102 (-0.894; 1.097)	-0.314 (-0.943; 0.314)	-0.489 (-0.939; 0.039)	0.014	1.199	1.474
Beliefs (M3)	0.025 (0.001; 0.051)	0.121 (-0.363; 0.604)	-0.540 (-0.845; -0.235)**	-0.164 (-0.382; 0.055)	0.050	3.835**	1.648
Motivation (M4)	-0.020 (-0.071; 0.030)	0.451 (-0.478; 1.381)	0.206 (-0.381; 0.792)	-0.282 (-0.702; 0.138)	0.011	1.306	1.683
External (M5)	-0.090 (-0.175; 0.005)	1.980 (0.410; 3.550)**	-0.095 (-1.086; 0.896)	-0.621 (-1.330; 0.089)	0.045	3.602**	1.391
Motivational Factors							
Disease prevention (M6)	0.001 (-0.016; 0.018)	0.183 (-0.127; 0.494)	-0.179 (-0.375; 0.017)	0.204 (0.064; 0.344)**	0.062	4.670**	1.621
Physical condition (M7)	-0.010 (-0.031; 0.010)	0.171 (-0.202; 0.544)	-0.074 (-0.310; 0.161)	0.336 (0.167; 0.504)***	0.082	5.945***	1.460
Weight control (M8)	0.009 (-0.008; 0.027)	0.263 (-0.053; 0.580)	-0.139 (-0.338; 0.061)	0.210 (0.067; 0.353)**	0.055	4.243**	1.763
Appearance (M9)	-0.008 (-0.027; 0.012)	0.292 (-0.072; 0.655)	-0.120 (-0.349; 0.110)	0.331 (0.167; 0.496)***	0.093	6.680***	1.718
Stress control (M10)	-0.009 (-0.019; 0.017)	0.236 (-0.100; 0.572)	-0.143 (-0.356; 0.069)	0.258 (0.106; 0.410)***	0.069	5.120***	1.812
Fun (M11)	-0.009 (-0.027; 0.009)	0.299 (-0.032; 0.631)	-0.054 (-0.263; 0.155)	0.259 (0.109; 0.409)***	0.067	4.960***	1.657
Affiliation (M12)	-0.013; (-0.035; 0.008)	0.275 (-0.128; 0.679)	-0.188 (-0.443; 0.066)	0.215 (0.032; 0.397)*	0.050	3.950***	1.495
Health rehabilitation (M13)	-0.010 (-0.034; 0.013)	0.310 (-0.123; 0.744)	-0.057 (-0.330; 0.217)	0.292 (0.096; 0.488)**	0.044	3.579 **	1.693
Competition (M14)	-0.018 (-0.040; 0.003)	0.471 (0.073; 0.869)*	-0.164 (-0.415; 0.087)	0.248 (0.069; 0.428)**	0.077	5.648***	1.760
Social recognition (M15)	-0.016 (-0.037; 0.005)	0.046 (-0.337; 0.430)	-0.085 (-0.328; 0.157)	0.323 (0.149; 0.496)***	0.081	5.897***	1.570

Note. Only unstandardized regression coefficients that were less than the 0.05 significance level are highlighted in bold. B = Unstandardized regression coefficient; CI = 95% confidence interval *p < 0.05. **p < 0.01. ***p < 0.001

In contrast to the barriers, all 10 regression models for the motivational factors (M6 to M15) were statistically significant (*p*<0.01), as shown in Table 5. The most robust and consistent predictor across all models was the weekly duration of exercise. This variable demonstrated a positive and highly significant association with all ten motivational factors investigated. For example, a higher weekly exercise duration predicted higher levels of motivation for Physical Condition (M7) (B = 0.336, 95% CI [0.167, 0.504], *p*<0.001), Appearance (M9) (B = 0.331, 95% CI [0.167, 0.496], *p* < 0.001), and Social Recognition (M15) (B = 0.323, 95% CI [0.149, 0.496], *p* < 0.001). The remaining variables had limited predictive power for motivation. Gender was a significant predictor only for the Competition (M14) factor (B = 0.471, 95% CI [0.073, 0.869], *p* < 0.05), suggesting that men are more motivated by competition than women. Age and weekly frequency of exercise were not significant predictors for any of the motivational factors. In summary, the findings suggest that exercise duration is a crucial factor in motivating older adults. In contrast, exercise frequency and demographic factors play more specific roles, primarily in mitigating certain perceived barriers to exercise.

While the regression models provided relevant findings into the individual predictors of exercise barriers and motivational factors, a deeper understanding of the interplay between motivation and perceived barriers, and how these factors coalesce within distinct subgroups, necessitated further exploration. Therefore, to identify homogeneous groups of older adults based on their motivational profiles and perceived barriers, a non-hierarchical cluster analysis was subsequently performed.

**Fig. 1:**
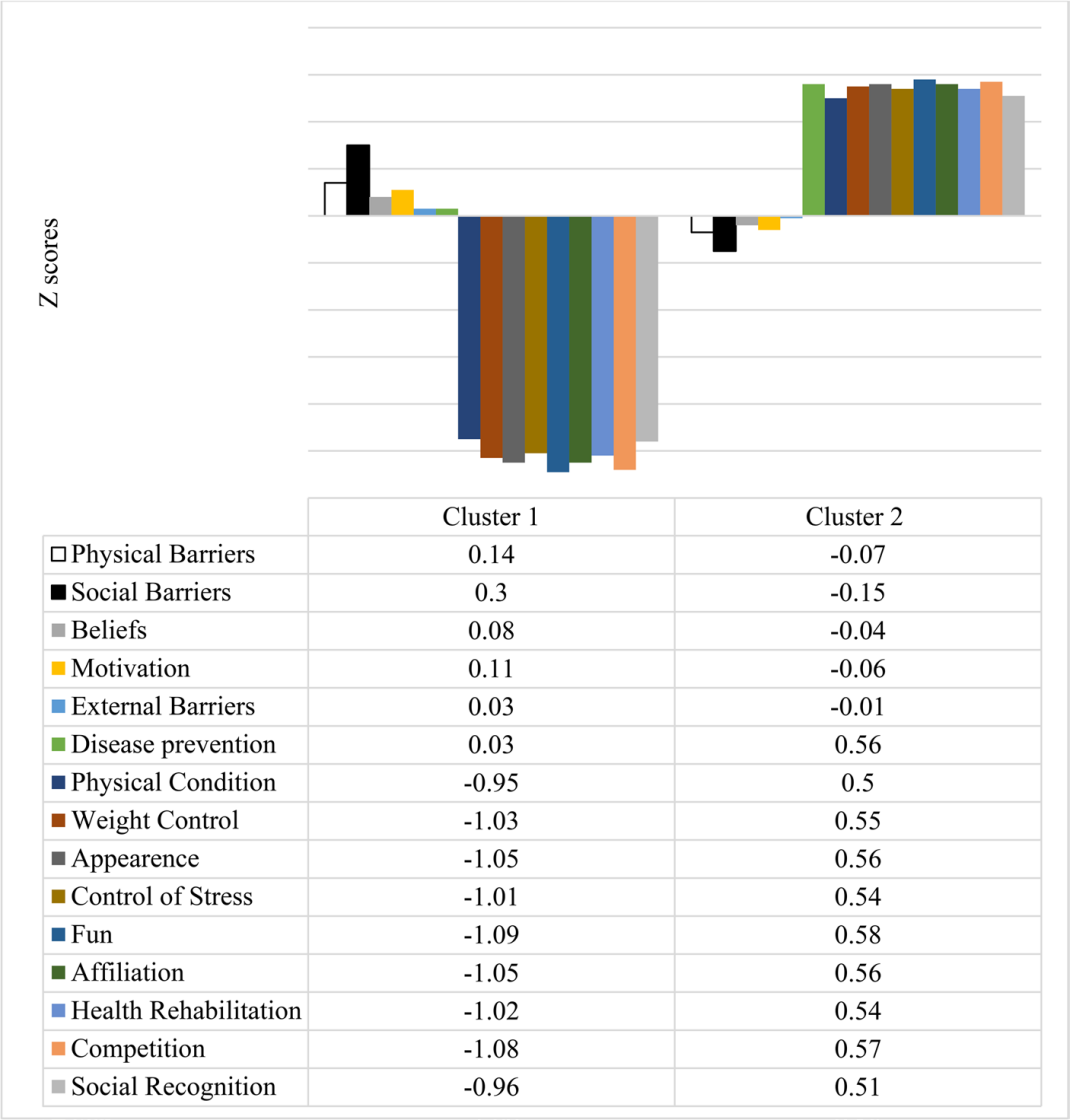
Graphical representation of the perception profiles of barriers and motivational factors for physical exercise among older adults through cluster analysis

This analysis confirmed a two-cluster solution, as described in Fig. 1. Older adults in Cluster 1 (*n* = 78) showed low motivation and moderate perception of barriers, and they were labeled “Low motivation and moderate perception of barriers.” Cluster 2 (*n* = 147) was characterized by high motivation scores and a low perception of barriers, and was labeled as “High motivation and low perception of barriers.”

In the association between profiles of barrier perception and motivational factors according to physical exercise practice variables (Table 6), a significant association was found only with the weekly time spent exercising at the location (*p* = 0.002). This association indicates a trend in which older adults with high motivation and low perception of barriers reported practicing more hours of physical exercise per week.

**Table 6:** Association between older adults' perceptions of barriers and motivation profiles according to exercise practice variables

VARIABLES	Cluster profile		X^2^	p-value
	Low motivation and moderate perception of barriers(n=78)	High motivation and low perception of barriers(n=147)		
	ƒ (%)	ƒ (%)		
Place of physical exercise practice				
Gym	20 (25.6)	55 (37.4)	1.436	0.231
SFA	31 (39.7)	44 (29.9)		
Sports Center	27 (34.6)	48 (32.7)		
Weekly frequency of exercise practice				
Daily	30 (38.5)	69 (46.9)	2.579	0.108
Weekly	41 (52.6)	72 (49.0)		
Rarely	7 (9.0)	6 (4.1)		
Weekly time spent practicing PE at the location				
Less than 1 h	18 (23.1)	18 (12.4)	9.585	0.002*
1 to 3 h	41 (52.6)	58 (40.0)		
3 to 5 h	14 (17.9)	56 (38.6)		
More than 5 h	5 (6.4)	13 (9.0)		

*Significant association – p < 0.05: Chi-square test

SFA: Senior Fitness Academies

## Discussion

This study revealed that older adults tend to perceive external and physical barriers to physical activity and are primarily motivated by health-related extrinsic goals, especially weight control. Regression models indicated that higher weekly frequency of exercise was associated with lower perception of barriers related to limiting beliefs, while women reported higher external barriers compared to men. Notably, weekly exercise duration emerged as the strongest and most consistent predictor of motivation across all assessed factors, suggesting that spending more time exercising is central to sustaining motivation in this population. Gender and frequency of exercise played more specific roles, primarily influencing certain barrier perceptions and the competition-related motivation factor, respectively. Overall, individuals with lower exercise engagement reported lower motivation and higher barrier perception, particularly concerning stress management, appearance, and affiliation.

From a theoretical perspective, these patterns are well explained by Self-Determination Theory (Ryan & Deci, 2020), which emphasizes the importance of satisfying the psychological needs for autonomy, competence, and relatedness in promoting autonomous motivation. Older adults who exercise less frequently may not experience sufficient positive reinforcement—such as improved physical capacity, mood, or social connection—which are necessary to satisfy these needs and transition from externally regulated behavior (e.g., exercising only for weight control) to more intrinsic or integrated forms of motivation (Ladeira et al., 2019). Conversely, those with more consistent routines likely benefit from this process, sustaining long-term engagement through a self-reinforcing cycle of competence and autonomy (Oliveira et al., 2019).

Environmental context also played a critical role. Despite professional supervision, participants from public sports centers reported higher perceptions of physical and social barriers. These findings suggest that structural limitations (e.g., outdated equipment, overcrowded facilities, or lack of personalization) may undermine feelings of competence and autonomy (Teixeira et al., 2012). In contrast, participants from private gyms reported higher motivation for competition, a marker of extrinsic but goal-oriented engagement. While this may reflect a more privileged socioeconomic background—an unmeasured confounder—it also suggests that resource-rich environments can provide structured pathways to foster competence, even through extrinsic channels (Deci & Ryan, 2000).

Interestingly, Senior Fitness Academies—public, unsupervised, open-air facilities—seemed to promote adherence by reducing logistical and psychological barriers. Their accessibility, low cost, and intuitive design may support autonomy and reduce pressure, especially for those with limited financial resources or social confidence. These findings suggest that autonomy-supportive, flexible environments—whether supervised or not—can play a crucial role in sustaining motivation, particularly when they are inclusive and easily accessible (Teixeira et al., 2012; Wells et al., 2024).

The two motivational profiles identified in the cluster analysis reinforce these theoretical assumptions. The profile characterized by high motivation and low perceived barriers was associated with greater weekly exercise volume, suggesting a virtuous cycle in which motivation and behavior are mutually reinforcing. In contrast, the low-motivation profile with moderate barrier perception was associated with less frequent engagement, possibly due to unmet psychological needs or lack of contextual support.

These findings offer practical insights for the design of public programs and policy. First, physical activity initiatives targeting older adults should avoid generic approaches. Instead, they should consider individual motivational profiles and adapt strategies accordingly. For instance, programs aimed at low-motivation individuals could emphasize short-term, tangible benefits and gradual skill-building to enhance competence and self-efficacy (Ryan & Deci, 2017). On the other hand, environments with stronger social and competitive dynamics might better serve those with more autonomous or performance-driven motivations (Camp et al., 2024).

Public infrastructure should also support autonomy, particularly by ensuring accessibility, safety, and minimal entry barriers (financial or logistical). Training and deploying qualified professionals in public settings can help strengthen feelings of competence and relatedness, especially when programs are personalized and supportive rather than prescriptive (WHO, 2018). These considerations are especially relevant for policymakers, as they reflect low-cost strategies that can be integrated into community health promotion (Bauman et al., 2016).

From a theoretical standpoint, the study contributes to SDT-based research by showing how diverse physical activity contexts influence the satisfaction (or frustration) of core psychological needs in older adults. It also demonstrates that motivation is a profoundly individual trait shaped by environmental design and access (Ryan & Deci, 2017; Teixeira et al., 2012; Markland et al., 2005).

The finding that higher weekly exercise frequency is associated with lower perception of barriers related to limiting beliefs suggests that regular practice can help reduce self-sabotaging thoughts or misconceptions about physical activity, such as “I am not capable,” “exercise is not for my age,” or “I will get injured.” This result is consistent with previous studies, which have shown that repeated practical experience with exercise contributes to increased self-efficacy (McAuley et al., 2011; Bauman et al., 2016). The older adults who practice more build confidence in their abilities, demystify negative beliefs, and begin to perceive exercise as a feasible and beneficial activity (Zemancová et al., 2022; Xie et al., 2025).

Moreover, regular frequency can act as positive reinforcement, reducing fear and insecurity while reinforcing realistic perceptions of one’s functional capacity. This highlights the importance of adherence strategies that promote regularity and consistency, even if at lower volume or intensity (Wells et al., 2024).

On the other hand, the finding that women perceive more external barriers than men (such as lack of safety, absence of companionship, or lack of adequate environments) reflects well-documented cultural and social issues. Studies (Baert et al., 2015; Ciaccioni et al., 2025) indicate that older women often face greater challenges related to social and environmental contexts, including public safety concerns, gender norms that limit their participation in physical activities, and increased workload from domestic or caregiving responsibilities.

This heightened awareness of external barriers may negatively impact adherence and reinforces the need for targeted interventions, such as offering programs in safe locations, women-only groups, and community initiatives that foster social support networks. Additionally, interventions that consider the social and family realities of older women are essential to overcoming these barriers and facilitating engagement.

The finding that weekly exercise duration was the most robust and consistent predictor for all motivational factors reinforces the importance of total practice volume as a central element in sustaining motivation among older adults. The positive and significant association with factors such as physical condition, appearance, and social recognition indicates that the more time older adults dedicate to physical activity throughout the week, the greater their perceived benefits and, consequently, their motivational engagement.

This result aligns with SDT, which suggests that longer and more regular practice strengthens the internalization of motives for exercise, shifting individuals from extrinsic motivations (e.g., appearance or social pressure) toward more intrinsic motivations (e.g., pleasure, personal satisfaction, health improvement) (Deci & Ryan, 2000; Teixeira et al., 2012).

The absence of a significant effect of age and weekly frequency on motivation is also noteworthy. These findings indicate that, for older adults, simply exercising on more days does not necessarily guarantee greater motivation; instead, the total time dedicated seems to be determinant for experiencing and perceiving benefits, thereby strengthening the motives to continue.

Additionally, the fact that sex emerged as a significant predictor only for the competition factor suggests a differentiated motivational profile between men and women. The greater importance attributed to competition among men has been described in previous studies and may be related to sociocultural values and gender socialization, which have historically associated men with more competitive behaviors (Molanorouzi et al., 2015).

This study has limitations. The sample size (*n* = 75 per group) was modest, and convenience sampling limits the generalizability of the findings. Although the equal allocation across the three groups allowed for balanced comparison between different exercise environments, it does not reflect the natural population distribution of users in these settings. As a result, differences in sociodemographic characteristics—such as age, education, and income—may have influenced the results and acted as potential confounders. Additionally, the cross-sectional design precludes causal inference, and self-reported data may have introduced recall or social desirability bias. Variables such as socioeconomic status, pre-existing health conditions, and availability of social support were not included in the analysis but should be considered in future studies. The exclusion of participants who trained with personal trainers also limited our understanding of more individualized exercise contexts. Finally, the predominance of women in the sample reflects demographic trends in community-based exercise programs but restricts the exploration of sex-based differences.

Future studies should incorporate longitudinal and mixed-methods approaches to explore motivational dynamics over time and deepen the understanding of how older adults experience environmental facilitators and constraints. Incorporating confounding variables such as SES, comorbidities, and access to services will also allow for more targeted and equitable recommendations. Importantly, translating SDT-based findings into scalable policy and programmatic interventions remains a key avenue for applied research.

## Conclusion

It is concluded that barriers and motivations for engaging in physical exercise among older adults vary according to the exercise setting, weekly frequency, and time dedicated. Social and motivational barriers negatively affect adherence, while health-related beliefs may serve as incentives. Older adults attending public sports centers reported more barriers and lower motivation, whereas those in private gyms showed higher motivation for competition.

Notably, the regression analyses revealed that higher weekly exercise duration is the most robust predictor of motivation across all investigated factors, emphasizing its central role in sustaining engagement. Furthermore, higher weekly exercise frequency was associated with lower perception of barriers related to limiting beliefs, suggesting that consistent practice contributes to reducing negative cognitions. Gender differences were also evident, with women perceiving more external barriers and men being more motivated by competition.

Cluster analysis further supported these patterns, showing that higher motivation and lower perceived barriers are consistently associated with greater exercise engagement.

These findings highlight the need for context-sensitive interventions that not only reduce barriers but also prioritize strategies to increase exercise duration and address gender-specific needs, thus promoting more accessible, supportive, and motivating environments for older adults.

## Data Availability

No datasets were generated or analysed during the current study.
